# Supplementary material for: Elective Ascending Aortic Aneurysm Surgery in the Elderly
Source: J Clin Med. 2023 Mar 3;12(5):2015. doi: 10.3390/jcm12052015 (PMC10004422; doi:10.3390/jcm12052015)
Supplement: Supplementary file 1 [file jcm-12-02015-s001.zip › jcm-2223149-supplementary.pdf]

Supplementary Material

Supplemental File S1. Performed Surgical Techniques in this Study

During the inclusion period, there was a substantial variability in the applied surgical techniques. Below we describe the most commonly used surgical procedures are described here. The surgical interventions were performed using a median sternotomy approach. After heparinization, cardiopulmonary bypass (CPB) was initiated which was followed by aortic clamping. Due to the elective character of the surgical interventions, it was in most cases possible to cannulate in the ascending aorta. However, the aortic arch, femoral artery and subclavian artery were used as well. Venous cannulation was performed by using a two-stage cannula in the right atrium and inferior vena cava, or cannulation in the superior and inferior vena cava or in the femoral artery. Myocardial protection was performed with the use of cold cardioplegia. If deep hypothermic cardiac arrest (DHCA) was performed, patients were cooled to a nasal temperature of 20 or 25 °C nasal until pO_2 was 40 mmHg. In most cases, uni- or bilateral antegrade cerebral perfusion (ACP) was used, especially when a long DHCA time (>30 minutes) was expected. Indications for DHCA include congenital aortic arch surgery and heavy aortic calcification since distal aortic clamping would increase stroke risk. In the final years of the study, bilateral ACP was mainly used, with cooling down to below 25 degrees. When DHCA was not performed, a temperature of 32 to 35 degrees was maintained for the moderate hypothermia.

Depending on the indication, and surgeon and patient preference, Bentall(- de Bono) (1), Valve Sparing Aortic Root Replacement (VSARR) or Supracoronary Aorta Replacement (SCAR) were performed.

In the Bentall procedure, the mechanical Bentall was a ready-made tube plus valve in one. The biological Bentall was either constructed in the operating room or was a ready-made

compound conduit. Firstly, the enlarged portion of the ascending aorta and the aortic valve were explanted and the coronary buttons were prepared, placing U-stitches around. The graft with valve was replaced and then an edge of aortic tissue was adhered to the prosthesis for hemostasis. Hereafter, the coronary buttons were reimplanted. Finally, an end-to-end anastomosis between prosthesis and native distal aorta was performed.

The VSARR included the David (or reimplantation) (2) and Yacoub (or remodeling) (3) techniques. In the David technique, the coronary buttons were prepared and the aortic wall was excised. The stitches were placed on felt. Then, the stitches were pulled through the base of the prosthesis and tied off. The valve was reimplanted in the prosthesis. The edges of aortic tissue were then adhered to the prosthesis, with anchoring of the commissures. Finally, end-to-end anastomosis of the graft to the distal aorta was performed.

Two Yacoub techniques were performed during the study period: the classic Yacoub and the partial Yacoub technique, which was performed in most of the patients. In the Yacoub technique, the annulus of the aorta was not involved in the procedure compared to the David technique. Furthermore, the partial Yacoub procedure consisted of the excision of the non-coronary sinus.

Finally, SCAR was also performed in the study cohort. The aorta was excised above the sinuses of Valsalva, and the graft was anastomosed to the aortic root, after which an end-to-end anastomosis of the graft to the distal aorta was performed.

(1) Bentall H, De Bono A. A technique for complete replacement of the ascending aorta. Thorax. 1968;23(4):338-9.

(2) David TE, David CM, Manlhiot C, Colman J, Crean AM, Bradley T. Outcomes of Aortic Valve-Sparing Operations in Marfan Syndrome. J Am Coll Cardiol. 2015;66(13):1445-53.

(3) Yacoub M. (1996). Valve-Conserving Operation for Aortic Root Aneurysm or Dissection. Operative Techniques in Cardiac & Thoracic Surgery, Vol 1, No 1 (July), 1996: pp 57-67.

Supplemental File S2. Definitions of all included variables.

Variable	*Unit*	*Definition*
Patient Characteristics		
Year of birth		Year of birth
Age at time of surgery	years	
Sex	none	Sex: male or female
Height	meters	At admittance or last measured before surgery
Weight	kilograms	At admittance or last measured before surgery
Body surface area	m^2	At admittance or last measured before surgery
Body mass index	kg/m^2	At admittance or last measured before surgery
Prior aortic surgery		Has the patient had aortic surgery before? Options: same part of aorta, other part of aorta, same and other part of aorta, other and unknown.
Description of prior aortic surgery		Short description of prior aortic surgery including date of prior surgery
Prior cardiac surgery		Has the patient had cardiac surgery before? Options: CABG, PTCA, AVR, CABG+AVR, other and unknown.
Description of prior cardiac surgery		Short description of prior cardiac surgery including date of prior surgery
Recent myocardial infarction		(N)STEMI < 90 days of presentation
History of myocardial infarction		(N)STEMI in patient history: > 90 days before presentation
History of aortic valve stenosis		Is the patient known with aortic valve stenosis? Trace= grade 1, slight= grade 2, moderate= grade 3, severe= grade 4. Moderate aortic valve stenosis: V_{max} 3.0-3.9 m/s and AVA 1.1-1.5 cm^2, and severe stenosis: V_{max} ≥4.0 m/s and AVA ≤1.0 cm^2.
History of aortic valve insufficiency		Is the patient known with aortic valve insufficiency? Trace= grade 1, slight= grade 2, moderate= grade 3, severe= grade 4. Moderate aortic valve insufficiency: jet width 25-64% of the left ventricular outflow tract (LVOT), and severe insufficiency: ≥65% of LVOT.
Mixed aortic valve disease		Mixed disease is defined as stenosis and regurgitation

History of mitral valve stenosis	Is the patient known with mitral valve stenosis? Trace= grade 1, slight= grade 2, moderate= grade 3, severe= grade 4
History of mitral valve insufficiency	Is the patient known with mitral valve insufficiency? Trace= grade 1, slight= grade 2, moderate= grade 3, severe= grade 4
History of hypertension	Medical treatment for hypertension or recorded in patient history
History of diabetes mellitus	Medical treatment for diabetes mellitus or recorded in patient history. Options: NIDDM, IDDM and unknown.
History of COPD	History of COPD that required medical treatment or when FEV1 was <70%
History of CVA	TIA and/or CVA in patient history
History of chronic kidney disease	Is the patient known with chronic kidney disease? Yes when this is recorded in patient history. Thus, it is not dependent on the current eGFR.
History of renal dialysis	The patient is currently receiving any type of renal dialysis for chronic kidney disease
History of smoking	From at least 1 packyear. Options: never, currently, in past and unknown.
History of hyperlipidaemia	Medical treatment for hyperlipidaemia/dyslipidaemia/ hypercholesterolemia or recorded in patient history
Prior dissection or aneurysm in a major artery other than the thoracic aorta?	Is the patient familiar with a dissection elsewhere (every vessel except the thoracic aorta)?
Known genetic disease prior to surgery?	Any genetic disorder confirmed by genetic testing, if not confirmed then: suspected. Options: Marfan syndrome, Loeys-Dietz syndrome, SMAD3 gene mutation, Ehlers-Danlos syndrome, ACTA 2 gene mutation, MYH11 mutation, Turner syndrome, et cetera.
Is there a positive family history of aortic pathology?	1st or 2nd degree family member with: thoracic aortic aneurysm or dissection; an aneurysm or dissection elsewhere < 60 y; congenital left sided heart disease or sudden death <45y.
Is there a positive family history of any connective tissue disorder?	Any connective tissue disorder in 1st or 2nd degree family members

Remarks patient characteristics		Any remarks regarding patient characteristics
Pre-operative		
How did the patient present to the clinic for the very first time?		How was the aortic aneurysm discovered? Options: coincidental finding, (family) screening, (suspected) connective tissue disorder, exercise related symptoms/complaints, non-exercise related symptoms/complaints and unknown.
At which location was the maximal diameter of the aorta measured?		Location of the maximal aortic diameter. Options: sinuses of Valsalva, ascending aorta, aortic arch, descending aorta, abdominal aorta and unknown.
Maximal aortic diameter measured before surgery	millimetres	Measured diameter of maximal aortic diameter
Dilatation of aortic valve annulus		As described in the patient files (>40 mm)
Dilatation of Sinuses of Valsalva		As described in the patient files (>40 mm)
Dilatation of ascending aorta		As described in the patient files (>40 mm)
Dilatation of aortic arch		As described in the patient files (>40 mm)
Dilatation of descending aorta		As described in the patient files (>40 mm)
Dilatation of abdominal aorta		As described in the patient files (>30 mm)
Does the aortic aneurysm extend into the brachiocephalic artery?		Does the patient has dilatation/aneurysm of the brachiocephalic artery at the same time?
Does the aortic aneurysm extend into the carotid artery?		Does the patient has dilatation/aneurysm of the carotid artery (left and/or right) at the same time?
Does the aortic aneurysm extend into the subclavian artery?		Does the patient has dilatation/aneurysm of the subclavian artery at the same time?

Intramural hematoma present before surgery?		Intramural hematoma of the aorta present?
Intramural ulcer present before surgery?		Intramural ulcer of the aorta present?
NYHA classification at presentation		As described in patient files. Options: NYHA class I, NYHA class II, NYHA class III, NYHA class IV and unknown.
CCS classification at presentation		As described in patient files. Options: CCS I, CCS II, CCS III, CCS IV and unknown.
Glomerular filtration rate	ml/min	Measured at admittance, or maximum 1y before surgery. For the study, only the CKD-EPI eGFR measurement was used.
Date of last eGFR measurement before surgery	dd-mm-yyyy	Date of last eGFR measurement was performed before surgery
Creatinin-level	micromol/L	Measured at admittance, or maximum 1y before surgery
Date of last creatinin measurement before surgery	dd-mm-yyyy	Date of last creatinin measurement was performed before surgery
Left ventricular ejection fraction		Last measured before surgery. Options: Good (>55%), reduced (45-55%), moderate (30-45%), poor (30%) and unknown.
Logistic EUROscore		As described in patient files
Heart rhythm registered on electrocardiogram		As described in patient file or as reported by computer on ECG itself. Options: sinus rhythm (60-100 bpm), sinus tachycardia (>100 bpm), sinus bradycardia (<60 bpm), supraventricular tachycardia (incl: atrial fibrillation/atrial flutter), paced rhythm (atrial and/or ventricular pacing), ventricular tachycardia / fibrillation, other (describe below) and unknown.
Explain 'other' rhythm seen on ECG		Other rhythm as described in patient file
Any signs of ischemia on ECG?		As described in patient file or as reported by computer on ECG itself
Remarks on pre-operative status patient		Any important details on pre-operative variables not yet specified
Imaging: transthoracic echocardiography, transoesophageal echocardiography, computed tomography (CT) and magnetic resonance imaging (MRI)		
Date	dd-mm-yyyy	Date of the performed imaging

Annulus diameter	millimetres	As described in imaging report
Sinus of Valsalva diameter	millimetres	As described in imaging report
ST-junction diameter	millimetres	As described in imaging report
Ascending aorta diameter	millimetres	As described in imaging report
Arch diameter	millimetres	As described in imaging report
Descending aorta diameter		As described in imaging report
Aortic valve stenosis		Is the patient known with aortic valve stenosis? Trace= grade 1, slight= grade 2, moderate= grade 3, severe= grade 4
Aortic valve insufficiency		Is the patient known with aortic valve insufficiency? Trace= grade 1, slight= grade 2, moderate= grade 3, severe= grade 4
Aortic valve mixed disease		Mixed disease is defined as stenosis and regurgitation
Mitral valve stenosis		Is the patient known with mitral valve stenosis? Trace= grade 1, slight= grade 2, moderate= grade 3, severe= grade 4
Mitral valve insufficiency		Is the patient known with mitral valve insufficiency? Trace= grade 1, slight= grade 2, moderate= grade 3, severe= grade 4
Measurement technique LVEF		Technique used to measure LVEF. Options are: M-mode, Quinones, Biplane (Modified Simpson), WMSI, or unknown.
LV(E)F	%	As described in imaging report
Other abnormalities		Other abnormalities regarding imaging of the aorta
Remarks		Any remarks regarding imaging of the aorta
Operative		
Urgency of operation		(Duration from diagnosis until surgery). Options: acute (<24 hours), urgent (within 2 weeks or during hospital stay), elective (>2 weeks) and unknown.
Operation date		The date on which the aortic surgery was performed
Incision location		Location as described in surgery report. Options: sternotomy, thoracotomy, thoracotomy and laparotomy, other and unknown.
ECC technique		Technique used for ExtraCorporeal Circulation as described in surgery report. Options: none, passive shunt, left heart bypass, full bypass and unknown.
ECC cannulation arterial		Location of arterial ExtraCorporeal Circulation placement as described in surgery report. Options: none, femoralis, subclavia, aorta ascendens, aortic arch, other and unknown.

Description of other arterial ECC cannulation		As described in surgery report
ECC cannulation venous		Location of venous ExtraCorporeal Circulation placement as described in surgery report. Options: none, vena cava superior, vena cava inferior, vena cava superior and inferior, right atrium, femoralis, other and unknown.
Description of other venous ECC cannulation		As described in surgery or anaesthesia report
Perfusion time	minutes	As described in surgery or anaesthesia report
Cardiac ischemic time	minutes	As described in surgery or anaesthesia report
Aortic ischemic time	minutes	As described in surgery or anaesthesia report
Myelum ischemic time	minutes	As described in surgery or anaesthesia report
Circulatory arrest time	minutes	As described in surgery or anaesthesia report
Cerebral perfusion type	minutes	As described in surgery or anaesthesia report. Options: none, antegrade unilateral, antegrade bilateral, retrograde and unknown.
Cerebral perfusion time	minutes	As described in surgery or anaesthesia report
Deep hypothermic cardiac arrest (DHCA) used?		As described in surgery or anaesthesia report
Lowest temperature	degrees Celsius	As described in anaesthesia report
Location of temperature measurement		As described in anaesthesia report
Time at start ECC	hh.mm	As described in anaesthesia report
Temperature at start of ECC	degrees Celsius	As described in anaesthesia report
Time at start DHCA	hh.mm	As described in anaesthesia report
Temperature at start of DHCA	degrees Celsius	As described in anaesthesia report
Time at end DHCA	hh.mm	As described in anaesthesia report
Temperature at end of DHCA	degrees Celsius	As described in anaesthesia report
Time at end ECC	hh.mm	As described in anaesthesia report
Temperature at end of ECC	degrees Celsius	As described in anaesthesia report
Sievers classification		Observed Sievers classification of the bicuspid aortic valve during surgery, as described in surgery report. Options: no BAV, type 0,

		type 1 LCC-RCC fusion, type 1 RCC-NCC fusion, type 1 NCC-LCC fusion, type 2 and unknown.
Aortic annulus surgery		Was aortic annulus surgery performed? Options: no, yes with ring and unknown.
Aortic valve surgery		Aortic valve surgery performed during aortic surgery. Options: none, repair, replacement with mechanical valve, replacement with bioprosthesis, replacement with homograft, replacement with allograft, replacement with mechanical valved conduit (mechanical Bentall), replacement with biological valved conduit (Bio-Bentall), valve sparing David (reimplantation), valve sparing Yacoub (remodeling), other or unknown.
Aortic valve graft size	millimetres	Graft size used during surgery
Aortic root graft size	millimetres	Graft size used during surgery
Ascending aorta surgery		Was ascending aorta surgery performed? Options: none, replacement, repair or other and unknown.
Ascending aorta graft size	millimetres	Graft size used during surgery
Aortic arch surgery		Was aortic arch surgery performed? Options: none, replacement, hemi-arch, repair/patch/other, elephant trunc and unknown.
Aortic arch surgery graft size	millimetres	Graft size used during surgery
Reimplantation aortic arch branches		Were reimplantation of aortic arch branches performed? Options: none, with patch, separately, en bloc and unknown.
Reimplantation brachiocephalica		Was reimplantation of the brachiocephalic artery performed?
Reimplantation left common carotid		Was reimplantation of the left common aortic performed?
Reimplantation left subclavian		Was reimplantation of the left subclavian performed?
Descending aorta surgery		Was descending aorta surgery performed? Options: none, replacement, repair/patch/other and unknown.
Reimplantation intercostal arteries		Was reimplantation of the intercostal arteries performed?

Reimplantation intercostal arteries number		As described in anaesthesia report
Thoracoabdominal aorta surgery		Was thoracoabdominal aorta surgery performed? Options: none, distal anastomosis suprarenal, distal anastomosis infrarenal, distal anastomosis beyond bifurcation and unknown.
Reimplantation renal arteries		Was reimplantation of the renal arteries performed?
Reimplantation visceral arteries		Was reimplantation of the visceral arteries performed?
Concomitant procedures		Other procedures than aortic aneurysm surgery, or as described before performed. Options: none, CABG, mitral valve surgery, CABG and mitral valve surgery, other and unknown.
Description of other concomitant procedures		As described in surgery report
Any remarks regarding operative variables		
Postoperative		
Numer of days the patient was admitted	days	Total time in days from admission until discharge
Date of discharge to home, other hospital or institute	dd-mm-yyyy	As described in patient files
Number of days in ICU after surgery	days	Number of days that the patient stayed in the intensive care unit after surgery
Number of days on ventilation support after surgery	days	Number of days that the patient was on ventilation support after surgery
Death within 30 days OR before hospital discharge		Mortality within 30 days or before hospital discharge. Options: no, yes pre-surgery, yes in operating theatre, yes post-surgery and unknown.
Cause of in hospital death		As described in patient files. Options: no in-hospital death, bleeding, cardiac (incl. tamponade), aortic rupture, organ failure, sepsis, neurologic, other and unknown.

Reoperation needed	Was reoperation performed? Options: no reoperation, bleeding, tamponade, mediastinitis, cardiac ischemia, other visceral ischemia, structural valve deterioration, non-structural valve dysfunction, other graft dysfunction, endocarditis, other and unknown.
Indication for reoperation	Indication of reoperation when performed
Implantation of tracheostoma postop?	Did the patient receive implantation of tracheostoma after surgery?
New permanent heart rythm disturbances after surgery?	If postoperative pacemaker or ICD implantation was performed, if medication is used to treat arrhythmia, or if it is named permanent in the patient files. Options: supraventricular, ventricular, AV-block or unknown
Pacemaker or ICD implanted after surgery?	Only when the pacemaker or ICD was implanted within 14 days after surgery. Options: no, yes pacemaker, yes ICD and unknown.
What was the indication for pacemaker implantation?	As described in patient files
What was the indication for ICD implantation?	As described in patient files
Myocardial infarction or ischemia after surgery	During admission or within 30 days after surgery
Other cardiac complications after surgery?	During admission or within 30 days after surgery
Explain 'other' cardiac complication after surgery	
Bleeding event after surgery	During admission or within 30 days after surgery
Explain bleeding event after surgery	During admission or within 30 days after surgery
Diagnosis of infective endocarditis after surgery?	During admission or within 30 days after surgery
Structural valve deterioration after surgery?	During admission or within 30 days after surgery. Structural valve deterioration was defined as a result of failure of the valve itself.
Describe type of structural valve deterioration:	

Non-structural valve dysfunction after surgery?		During admission or within 30 days after surgery. Non-structural valve disease was defined as not due to the fall of the valve itself resulting in stenosis, regurgitation or hemolysis. Valve thrombosis and infection are not included.
Describe type of non-structural valve dysfunction		
Valve thrombosis after surgery?		During admission or within 30 days after surgery
Left ventricular thrombus diagnosed after surgery?		During admission or within 30 days after surgery
Lowest eGFR measured during admittance after surgery?		During admission or within 30 days after surgery. For the study, only the CKD-EPI eGFR measurement was used.
Last eGFR-level measured before discharge		For the study, only the CKD-EPI eGFR measurement was used
Last creatinine-level measured before discharge	micromol/L	During admission or within 30 days after surgery
TIA after Surgery?		During admission or within 30 days after surgery
CVA diagnosed after surgery?		During admission or within 30 days after surgery
Spinal cord lesion after surgery		During admission or within 30 days after surgery
New recurrence nerve lesion after surgery		During admission or within 30 days after surgery
Other neurological complications?		During admission or within 30 days after surgery
Describe other neurological complications diagnosed.		During admission or within 30 days after surgery
Diagnosis of psychiatric disorder after surgery (e.g. delirium)?		During admission or within 30 days after surgery. Delirium also falls under this category.
Diagnosis of any infection after surgery?		During admission or within 30 days after surgery

Describe the type of infection(s) that occured after surgery	Superficial wound infection was defined as wound infections for which no reoperation has been performed. Deep wound infection was defined as wound infection for which reoperation has been performed.
Diagnosis of sepsis after surgery?	During admission or within 30 days after surgery
Occurence of any vascular complications (eg ischemia, thrombosis, compartment syndrome)	During admission or within 30 days after surgery
Describe the vascular complication that occured	
If any other post-surgery complications were registered (eg pulmonary embolism, pneumothorax, decubitus, urinary retention) please explain here:	During admission or within 30 days after surgery

Supplemental File S3. Extra-analyses for the ≥75 years patient population

Table S1. *Short-term Mortality and Admission Days after Elective Aortic Aneurysm Surgery*

	**Total (n= 955)**	**Aged <75 yrs (n= 849)**	**Aged ≥75 yrs (n= 106)**	***p*-value**	**Missings**
In-hospital or 30-day Mortality	18 (1.9)	14 (1.6)	4 (3.8)	0.13	0/955
Cause of Mortality					0/955
Cardiac (incl. Tamponade)	8 (0.8)	7 (0.8)	1 (0.9)	1.00	
Bleeding	3 (0.3)	2 (0.2)	1 (0.9)	0.30	
Aortic Rupture	2 (0.2)	2 (0.2)	0 (0.0)	1.00	
Organ Failure	1 (0.1)	0 (0.0)	1 (0.9)	0.11	
Sepsis	2 (0.2)	2 (0.2)	0 (0.0)	1.0	
Other	1 (0.1)	0 (0.0)	1 (0.9)	0.11	
Number of Days the patient was					0/955
Admitted[a]	23 (2.4)	21 (2.5)	2 (1.9)	1.00	
1-4	590 (61.7)	528 (62.2)	62 (58.5)	0.46	
5-9	206 (21.5)	183 (21.6)	22 (20.8)	0.85	
10-14	66 (6.9)	59 (6.9)	7 (6.6)	1.000	
15-19	71 (7.4)	58 (6.8)	13 (12.3)	**0.044***	
≥20	8.0 (7.0-	8.0 (7.0-	9.0 (7.0-	0.40	
Total[#]	11.0)	11.0)	13.0)		
Number of Days in ICU[1] after Surgery [a]					(23/849)/(1/106)
1-4	828 (86.6)	744 (90.0)	84 (80.0)	**0.002****	
5-9	71 (7.4)	57 (6.9)	14 (13.3)	**0.019****	
10-14	18 (1.9)	14 (1.7)	4 (3.8)	0.14	
15-19	5 (0.5)	4 (0.5)	1 (1.0)	0.45	
≥20	10 (1.0)	8 (1.0)	2 (1.9)	0.31	
Total[#]	2.0 (2.0-3.0)	2.0 (2.0-3.0)	2.0 (2.0-4.0)	**<0.001****	
Number of Days on Ventilation Support after Surgery [a]					(32/849)/(5/106)
1	552 (57.7)	512 (62.6)	39 (38.6)	**<0.001****	
2	308 (32.2)	257 (31.4)	51 (50.5)	**<0.001****	
3	19 (2.0)	17 (2.1)	2 (2.0)	1.00	
4	10 (1.0)	9 (1.1)	1 (1.0)	1.00	
≥5	31 (3.2)	23 (2.8)	8 (7.9)	**0.015****	
Total[#]	1.0 (1.0-2.0)	1.0 (1.0-2.0)	2.0 (1.0-2.0)	**<0.001****	

Continuous data are presented as mean ± SD when the distribution is normal, or median (Interquartile Range, IQR) for variables without normal distribution. Categorical data are presented as frequencies (percentages).

[#] *Presented as median (Interquartile Range, IQR) of the total number of days in the whole, nonelderly and elderly population*

ICU: Intensive Care Unit

[a]*Significant differences were mainly found in the outliers in the number of days in elderly patients.*

[1]*NE/E: Nonelderly/Elderly*

* Significant at the 0.05 level

** Significant at the 0.01 level

Table S2. *Short-term Postoperative Morbidity After Elective Aortic Aneurysm Surgery*

	**Total (n= 955)**	**Aged <75 yrs (n= 849)**	**Aged ≥75 yrs (n= 104)**	***p*-value**	**Missings**
Tracheostoma Implantation	8 (0.8)	6 (0.7)	2 (1.9)	0.22	(1/849)/(0/106)
New Permanent Heart Rhythm Disturbances	245 (25.7)	201 (23.7)	44 (41.5)	**<0.001****	(3/849)/(0/106)
Supraventricular	211 (22.1)	169 (19.9)	42 (39.6)	**<0.001****	
Ventricular	6 (0.6)	6 (0.7)	0 (0.0)	1.00	
AV-block	28 (2.9)	25 (2.9)	2 (1.9)	0.76	
Pacemaker or ICD Implanted	32 (3.3)	30 (3.5)	2 (1.9)	0.57	0/955
Myocardial Infarction or Ischemia	18 (1.9)	16 (1.9)	2 (1.9)	1.00	0/955
Other Cardiac Complications	69 (7.2)	57 (6.7)	12 (11.3)	0.086	(2/849)/(0/106)
Infective Endocarditis	4 (0.4)	3 (0.4)	1 (0.9)	0.38	(3/849)/(0/106)
Non-Structural Valve Dysfunction	2 (0.2)	2 (0.2)	0 (0.0)	1.00	0/955
CVA/TIA	43 (4.5)	38 (4.5)	5 (4.7)	0.81	(2/849)/(0/106)
New Recurrence Nerve Lesion	12 (1.3)	12 (1.4)	0 (0.0)	0.38	0/955
Other Neurological Complications	33 (3.5)	28 (3.3)	5 (4.7)	0.40	(1/849)/(0/106)
Diagnosis of Psychiatric Disorder	146 (15.3)	105 (12.4)	41 (38.7)	**<0.001****	0/955
Diagnosis of Infection	130 (13.6)	108 (12.7)	22 (20.8)	**0.023****	0/955
Pneumonia	60 (6.3)	49 (5.8)	11 (10.4)	0.065	
Superficial Wound Infection	15 (1.6)	12 (1.4)	3 (2.8)	0.23	
Severe Wound Infection	10 (1.0)	7 (0.8)	3 (2.8)	0.089	
Urinary Tract Infection	18 (1.9)	14 (1.6)	4 (3.8)	0.13	
Other	15 (1.6)	15 (1.8)	0 (0.0)	0.40	
Of Unknown Origin	25 (2.6)	20 (2.4)	(4.7)	0.19	
Diagnosis of Sepsis	11 (1.2)	9 (1.1)	2 (1.9)	0.35	0/955
Diagnosis of Vascular Complications	15 (1.6)	11 (1.3)	4 (3.8)	0.074	0/955

Continuous data are presented as mean ± SD when the distribution is normal, or median (Interquartile Range, IQR) for variables without normal distribution. Categorical data are presented as frequencies (percentages).

CVA/TIA: Cerebrovascular Accident/Transient Ischemic Attack

* Significant at the 0.05 level

** Significant at the 0.01 level

Supplemental File S4. Univariable analysis on factors associated with long-term survival

	Total (n=928)		*Elderly (n=227)*		*Nonelderly (n=701)*	
	HR (95% CI)	*p*-value	HR (95% CI)	*p*-value	HR (95% CI)	*p*-value
Baseline						
Male sex	0.51 (0.38-0.69)	**<0.001****	1.07 (0.69-1.64)	0.765	0.55 (0.35-0.86)	**0.009****
Age	1.08 (1.06-1.09)	**<0.001****	1.06 (0.99-1.13)	0.081	1.06 (1.04-1.09)	**<0.001****
BSA	0.21 (0.11-0.40)	**<0.001****	0.62 (0.21-1.87)	0.398	0.45 (0.18-1.13)	0.088
History of hypertension	2.73 (1.90-3.92)	**<0.001****	2.64 (1.21-5.78)	**0.015***	1.56 (0.99-2.45)	0.055
History of hyperlipidaemia	1.64 (1.20-2.23)	**0.001****	1.12 (0.74-1.72)	0.587	1.60 (1.01-2.53)	**0.045***
Diabetes	1.42 (0.82-2.46)	0.216	0.24 (0.06-0.97)	**0.045***	3.25 (1.75-6.03)	**<0.001****
COPD	1.94 (1.26-2.98)	**0.003****	1.13 (0.62-2.05)	0.694	2.13 (1.12-4.05)	**0.021***
Prior CVA	1.02 (0.64-1.63)	0.925	1.14 (0.65-1.99)	0.645	0.60 (0.24-1.48)	0.266
Prior MI	1.34 (0.66-2.70)	0.415	0.79 (0.26-2.40)	0.678	1.82 (0.74-4.51)	0.194
Chronic kidney disease	2.75 (1.66-4.56)	**<0.001****	1.85 (1.00-3.41)	0.051	2.20 (0.88-5.46)	0.091
Prior aortic surgery	0.48 (0.25-0.92)	**0.026***	0.78 (0.29-2.14)	0.631	0.57 (0.25-1.32)	0.188
Prior dissection or aneurysm in major artery other than thoracic aorta	0.52 (0.13-2.11)	0.362	1.13 (0.26-4.86)	0.868	0.05 (0.00-36.07)	0.369
BAV	0.65 (0.47-0.90)	**0.010***	0.76 (0.42-1.37)	0.366	1.10 (0.71-1.71)	0.675
Presentation						
GFR	0.97 (0.97-0.98)	**<0.001****	0.99 (0.98-1.00)	0.112	0.98 (0.97-0.99)	**<0.001****
Log Euroscore	1.04 (1.03-1.06)	**<0.001****	1.00 (0.98-1.02)	0.944	1.03 (1.01-1.06)	**0.017***
Surgery						
Perfusion time	1.00 (1.00-1.00)	0.178	1.00 (1.00-1.01)	0.102	1.00 (1.00-1.01)	0.443
AoX time	1.00 (1.00-1.00)	0.899	1.00 (1.00-1.01)	0.272	1.00 (1.00-1.01)	0.968
DHCA	1.72 (1.26-2.33)	**<0.001****	1.26 (0.81-1.95)	0.300	1.35 (0.87-2.11)	0.180
Aortic valve surgery	1.35 (0.94-1.93)	0.107	1.01 (0.63-1.60)	0.980	1.07 (0.59-1.94)	0.828
Aortic arch surgery	0.54 (0.40-0.74)	**<0.001****	0.68 (0.44-1.06)	0.085	0.74 (0.47-1.15)	0.182
Ascending aortic surgery	8.15 (2.02-32.85)	**0.003****	1.30 (0.18-9,37)	0.794	9.46 (1.32-68.00)	**0.026***
Supracoronary replacement	1.44 (1.06-1.96)	**0.018***	1.16 (0.76-1.77)	0.483	1.07 (0.68-1.70)	0.770
VSARR	0.23 (0.10-0.52)	**<0.001****	0.89 (0.28-2.84)	0.846	0.20 (0.06-0.64)	**0.006****
Concomitant procedures	2.05 (1.47-2.85)	**<0.001****	1.35 (0.83-2.20)	0.226	1.82 (1.15-2.89)	**0.011***

Data is presented as Hazard Ratio (95% percent confidence interval)

BSA= Body Surface Area; COPD: Chronic Obstructive Pulmonary Disease, CVA: Cerebrovascular Accident, MI: Myocardial Infarction, BAV= Bicuspid Aortic Valve; AA: Aortic Aneurysm, LVEF: Left Ventricular Ejection Fraction, HTAD: Hereditary Thoracic Aortic Disease, NYHA: New York Heart Association, GFR: Glomerular Filtration Rate, DHCA: Deep Hypothermic Cardiac Arrest, BAV: Bicuspid Aortic Valve, ICU: Intensive Care Unit
